# Intra-individual variation in performance on novel variants of similar tasks influences single factor explanations of general cognitive processes

**DOI:** 10.1098/rsos.171919

**Published:** 2018-07-11

**Authors:** Jayden O. van Horik, Ellis J. G. Langley, Mark A. Whiteside, Philippa R. Laker, Joah R. Madden

**Affiliations:** Centre for Research in Animal Behaviour, Psychology, University of Exeter, Exeter, UK

**Keywords:** general intelligence, cognition, test battery, pheasants

## Abstract

Intra-individual variation in performance within and across cognitive domains may confound interpretations of both domain-general and domain-specific abilities. Such variation is rarely considered in animal test batteries. We investigate individual consistency in performance by presenting pheasant chicks (*n* = 31), raised under standardized conditions, with nine different cognitive tasks. Among these tasks were two replicated novel variants of colour learning and colour reversal problems, tests of positional learning and memory, as well as two different tasks that captured multiple putative measures of inhibitory control and motor-related performance. These task variants were also used to compare subjects' performance on alternative test batteries comprised of different task combinations. Subjects’ performance improved with experience, yet we found relatively little consistency in their performance, both within similar tasks using different paradigms and across different tasks. Parallel analysis revealed non-significant factors when all nine tasks were included in a principal axis factor analysis. However, when different combinations of six of the nine tasks were included in principal axis factoring, 14 of 84 combinations revealed significant main factors, explaining between 28 and 35% of the variance in task performance. While comparable findings have been suggested to reflect domain-general intelligence in other species, we found no evidence to suggest that a single factor encompassed a diverse range of cognitive abilities in pheasants. Instead, we reveal how single factor explanations of cognitive processes can be influenced by test battery composition and intra-individual variation in performance across tasks. Our findings highlight the importance of conducting multiple tests within specific domains to ensure robust cognitive measures are obtained.

## 1. Introduction

Cognitive adaptations are considered to have evolved to solve particular domain-specific socio-ecological problems and provide a selective advantage if they enhance an individual's fitness [1–4]. One approach towards understanding cognitive evolution focuses on the selection pressures that drive individual differences in specific cognitive abilities. Yet some studies also reveal broader domain-general cognitive abilities in which selection may act upon [5]. In this case, performance in one domain is correlated with performance in other, unrelated, domains, even when the tests appear to have little in common with one another [6]. In such studies, performance across the test battery can be summarized by a single factor, termed ‘*g*’ (for ‘general intelligence’) typically accounting for around 40% of the total variation in task performance [5].

A ‘*g*’ factor has been reported in mammals: humans [7–10], non-human primates [11–13], mice, *Mus musculus* [14–19] and dogs [20]. It has also been reported in some studies of birds [21,22]. However, it remains unclear whether the underlying mechanisms that link performance across test batteries are similar across different species [5]. For example, it has been proposed that processes of associative learning may be more influential to test battery performance in non-human animals, whereas humans may be more likely to adopt rule-based approaches to solving cognitive tasks [23].

One problem in comparing the expression of ‘*g*’ across studies, especially across taxa, is that studies differ markedly in their experimental protocol and test battery design [24]. In non-human animals, test batteries may be constrained by the paradigms which an animal can and/or will interact with, and vary according to the facilities available to the researchers. Performance on cognitive tasks may be further influenced by lifelong enculturation with human artefacts and previous, either known or unknown, testing scenarios [25]. Consequently, the likelihood of detecting ‘*g*’, and the interpretation of what it may imply, is susceptible to the composition of the battery of tests deployed [24]. In humans, the construction of test batteries may assume the existence of a general factor, and hence exclusively include tests that are sensitive to it [26]. However, if all tests included in a battery use the same test paradigm, or rely on a single underlying process, such as associative learning, then the presence of a ‘*g*’ may be overstated or miss-ascribed to domain-specific processes [23,27]. Conversely, if the tests deployed are too disparate in their form, or processes that they reveal, then we may understate or fail to detect ‘*g*’. As such, it is necessary to design test batteries that capture specific, ecologically relevant cognitive abilities that are biomechanically plausible (e.g. a rat or pigeon can press or peck a lever, respectively, whereas a jellyfish may have more difficulty in performing the same action) and result in measurable outcomes that are more ‘mental’ than dispositional. While these present challenging objectives for animal studies, it has been possible to test whether different test batteries produce the same estimates of ‘*g*’ in humans [28,29]. The relationship between ‘*g*’ and test battery composition has been explored in some non-human animals, for example, in North Island robins [22] and apes [30], but requires further investigation. Addressing such questions requires evidence of independent and reliable psychometric test scores in non-human animals, with large sample sizes that are robust to low power. We may then determine whether covariance in test performance, which is interpreted as ‘*g*’, is simply an artefact of the suite of tests used, or a reliable indicator of an individual animal's domain-general intelligence.

We investigate how the detection of a single factor explanation of cognitive performance (*g*) depends on intra-individual variation in task performance and the composition of the test battery. Studies investigating ‘*g*’ in birds are typically constrained by small sample sizes (*n* = 11–20 subjects) and predominantly test males in the wild; in which the previous life experiences of their subjects are unknown [21,22,31]. Two recent studies have however used large sample sizes; one using 42 wild Australian magpies that were habituated to testing [32] and another using 49 captive wild-caught male swamp sparrows [33]. In the current study, we assess performances of 31 pheasants. We presented young (four to eight week old) male and female pheasants that we had reared under identical conditions from hatching with nine different tasks. This test battery assessed each individual's ability to learn to discriminate between rewarded and unrewarded colours and spatial locations (positional learning), remember the location of a concealed food reward (positional memory) and perform novel motor actions to access a reward. Performances on inhibitory control tasks were also assessed using a Detour Reach apparatus [34–36] and a reversal learning paradigm involving colour discriminations [37,38]. We consider these tasks to broadly encompass at least four different domains of cognition: associative learning, positional learning, motor cognition and executive function. We extend findings from those studies conducted on wild birds by including multiple tests within particular domains (i.e. colour discriminations involving two sets of novel colours, and two different motor-related tasks) to assess domain-specific intra-individual consistency in performance. We then investigate how the inclusion or the exclusion of different sets of tasks within a test battery influence the extraction and interpretations of single factor explanations of cognitive processes, i.e. ‘*g*’. Support for ‘*g*’ in pheasants may be revealed if performances across tasks, regardless of the composition of the test battery, are positively related, with a variety of diverse tasks contributing to a single factor that captures a meaningful amount of inter-individual variation in performance. Alternatively, an individual's performance on any one task may be unrelated to their performance on any other task. If so, then we would conclude that there is no underlying general ability that determines cognitive performance across different cognitive domains. Finally, we may find that performance on particular sets of tasks is related, but other tasks are unrelated to one another. If so, then we would conclude that rather than an individual exhibiting a single underlying (domain-general) cognitive ability, there may be several independent, but still basal (domain-specific) processes that link an individual's performance in sets of similar but not identical tasks. This is especially likely if the tasks that are related to one another are putatively considered to represent particular cognitive domains. The chance of detecting these three alternative outcomes is highly dependent on the exact composition of tasks incorporated in a test battery, so we explore, using analyses of subsets of tasks, how the choice of tasks affects the chances of detecting ‘*g*’, namely a single factor that is robust to parallel analysis.

## 2. Methods

### 2.1. Subjects

Two hundred pheasant chicks were hatched on the 27 May 2015 and randomly assigned to be reared in one of four identical aviaries (for housing, shaping and testing procedures, see [39,40]). Each chick received two testing sessions per day between 8 June and 24 July 2015 in which they voluntarily entered a testing arena and could interact with a given test apparatus while isolated from conspecifics. Within this arena, subjects were presented with nine cognitive tasks in a fixed order so that each individual was tested on the same task on the same day (see §2.2; table 1). Thirty-one individuals participated in all trials (a total of 290) of all nine tasks and were hence included in this study.

**Table 1.** Test battery tasks used to assess performance on each putative cognitive domain. The number of trials that each bird participated in was used to determine their performance measures (trials). Trials in parentheses are training trials that preceded test trials and were not used to determine performance.

putative domain	task	trials
motor skills	Paper Puncture	7
	Robo-Worm	1
positional ability	Positional Learning	50
	Positional Memory	(4+) 18
discrimination learning	Colour Learning 1	50
	Colour Learning 2	50
inhibitory control	Colour Reversal 1	50
	Colour Reversal 2	50
	Detour Reach	(4+) 1

### 2.2. Test battery

#### 2.2.1. Paper puncture

We adapted protocols, previously used on zebra finch [41], song-sparrows [34] and New Zealand robins [22], to capture performance on a task previously used to assess motor cognition, by determining how proficient individuals were when extracting a concealed mealworm food reward. Subjects were presented with an apparatus (45 × 15 cm) comprised of five wells (2 cm diameter × 1.8 cm deep) each containing a single live mealworm. One well contained a freely available mealworm that was not covered by crepe paper. The remaining four wells contained mealworms that were increasingly concealed by crepe paper at the following technical levels: (0) wells open, (1) wells 1/2 covered, (2) two cuts, (3) one cut and (4) no cut (fully concealed). The location of the different concealed wells was randomized across trials for all individuals. Subjects were presented with seven trials and we measured their improvement by plotting a linear regression of the trial number and the rank of the most difficult well opened on that trial as our response variable for each individual.

#### 2.2.2. Robo-Worm

We further assessed motor cognition by attaching a freshly killed mealworm to the second hand of a crystal-modulated electronic clock that was positioned horizontal to the floor. We then used an Arduino physical computing interface to override the crystal pulses of the second hand so that the worm moved in a series of jerky, sporadic, arcs which appeared random, but which were consistent for all birds. An additional baseline mealworm was placed at the front edge of the apparatus to standardize each subject's approach so that they had equal opportunity to observe the worm. Subjects were presented with one trial in which they could retrieve the moving worm. We used latencies from the consumption of the baseline mealworm to the consumption of the worm as our response variable for each individual.

#### 2.2.3. Colour learning and reversal learning

Subjects received two colour learning and reversal learning tasks involving novel colour discriminations. In the first task, subjects were required to discriminate between two colour-cued wells in which the contents were concealed by a layer of crepe paper. During this colour learning phase, one well was encircled by a green cue and contained a mealworm reward, while the other well was encircled by a blue cue and the contents were made inaccessible by covering it with hard black card placed under the crepe paper, which could not be pecked through. Subjects were allowed to make one choice per trial. A correct choice was scored if subjects first pecked into a rewarded well and an incorrect choice was scored if subjects first pecked into an unrewarded well. The location of the rewarded side was pseudorandomized across trials and did not occur on the same side for more than three consecutive trials. Subjects were presented with two sessions each containing ten trials (of pairs to discriminate) per day, one session in the morning and one in the afternoon and received a total of 50 trials. A similar protocol was conducted for the reversal trials, but this time the previously rewarded green well was no longer rewarded, and the previously unrewarded blue well became rewarded. Again, subjects received 50 trials on the reversal discrimination problem. Subjects were not trained to a standardized learning criterion during the colour learning discriminations before participating in the reversal learning discriminations. We instead standardized each individual's exposure to each discrimination problem (i.e. 50 trials), using similar procedures to Raine & Chittka [42]. Hence, these procedures do not permit us to quantify individual measures of inhibition that are independent of pre-reversal experience. After subjects experienced the green/blue learning and reversal tasks, they were presented with an identical learning and reversal discrimination problem involving novel colour cues, in which the learning phase rewarded well was associated with a yellow cue and the unrewarded well associated with a pink cue, and vice versa during the reversal phase. Hence, each subject experienced a total of 200 trials (50 trials on each of the four discrimination problems). Subjects experienced their first 10 reversal trials on the same day as their last 10 learning trials. To determine performance measures on the colour learning and reversal learning discriminations, we plotted a logistic curve based on the order of correct and incorrect choices an individual made across trials using R [43]. For each individual, we used their predicted trial number when the curve crossed the line as our criteria for the bird having learned the task, indicating that there was an 80% probability of the bird making a correct choice, as our response variable. We derived this measure by solving the equation *X* = (−ln0.25 – *b*_0_)/*b*_1_, where *b*_1_ is the slope of the learning curve, and *b*_0_ is the intercept.

#### 2.2.4. Positional learning

Procedures for the positional discrimination problem were identical to those of the colour discrimination problems, except that the wells were not cued by different colours, but instead the location of the reward was consistently in the top well (furthest from the chick) with the closer, bottom well being consistently unrewarded across the 50 trials. Response variables for each bird were determined by using a logistic curve (as above) and calculating the predicted number of trials each bird required to obtain a greater than 80% success of choosing correctly.

#### 2.2.5. Positional memory

Subjects were individually trained to locate a single well containing a mealworm reward from nine other unrewarded wells arranged in a 2 ×  5 cm grid. In the first four training sessions, all wells were uncovered and the location of the rewarded well remained constant. Subjects were then presented with 18 test trials, in which the contents of all of the 10 wells were concealed by opaque crepe paper. Again, the location of the rewarded well remained constant during these trials. We recorded the number of wells that each subject opened before locating the rewarded well. We then plotted a linear regression of the trial number and the number of errors committed as our response variable for each bird.

#### 2.2.6. Detour reach

Following Boogert *et al*. [34] and MacLean *et al*. [35], we initially presented subjects with a horizontally positioned opaque tube (6 cm diameter, 15 cm long) fixed to a base (20 × 20 cm). One freshly killed mealworm was positioned inside the centre of the tube and one was placed at each entrance of the tube. Each subject received four training trials in which they were required to place their head inside the opaque tube and retrieve the mealworm reward. Subjects were then presented with a single test trial in which the opaque tube was replaced with an identical, but transparent tube. During the test trial, however, only the single mealworm in the centre of the tube was present. There was also a baseline mealworm placed outside the cylinder at its middle, facing the approaching chick. This served to centre the chick and position it so that it could see the reward mealworm directly in front of it, separated by the transparent barrier. We recorded, as a measure of inhibitory control [35], the number of pecks each subject directed towards the reward during the single test trial, i.e. failed attempts to acquire the mealworm, before the subject placed its head inside the apparatus and retrieved the reward. Hence, individuals that made fewer redundant pecks to the apparatus before acquiring the reward were considered to possess greater capacities for inhibitory control.

#### 2.2.7. Sex and body condition

At 10 weeks old, after testing had ceased, all subjects were sexed and their mass was recorded using a spring balance scale (Slater Super Samsom—precision 5 g) and tarsus length measured using a calliper (precision 0.1 mm).

#### 2.2.8. Statistical analysis

All statistical analyses were conducted in SPSS [44]. We used repeated measures ANOVA to determine whether subjects' performances improved across trials and hence learned each task. Improvement was considered the proportion of correct choices in the first 10-trial session and the final 10-trial session of a given task. Improvement and task (Colour Learning, Reversal Learning and Positional Learning tasks) were included as factors in the analysis. First and final trials were used to assess improvements in performance for motor skills and positional memory tasks. We also used planned, uncorrected paired *t*-tests to further compare initial and final performance on each task independently. All performance scores, except those of the Paper Puncture task, were inversely transformed prior to analyses as successful performance was attributed to fewer errors, trials or lower latencies.

We performed principal axis factoring (PAF), using the first unrotated factor to investigate whether individual performance across tasks could be explained by a single factor. The decision to use PAF, rather than principal components analysis (PCA), which is often (incorrectly) used to assess performance on animal cognitive test batteries [24], is because PAF is more appropriate for investigating the latent structure of cognitive abilities [45]. PCA is a descriptive technique that can be used to simplify interpretations of relationships between a large number of variables (performances on different tasks) of unknown relationships. PCA analyses all the variance from each variable and adopts a two-directional approach to predict variables by components and vice versa. By contrast, PAF is an exploratory technique in which causal relationships between variables are assumed, which should hence load on to the same factor. PAF is a modelling method that analyses only shared variance between variables (i.e. leaves out unique variance), and hence latent factors steer observed variables one-directionally. PAF can therefore be used to illuminate whether the same cognitive ability underlies performance on different tasks that are presumed to be governed by the same cognitive process. For example, the same cognitive ability may be considered to underlie performance on multiple variants of tasks that use different colours to assess discrimination learning. Conversely, PCA could be used instead to reduce/simplify performances obtained from a number of different colour discrimination tasks, so that one variable represents an ability to discriminate between different colours. Task loadings greater than 0.4 were considered salient [46]. We used parallel analysis to assess the likelihood that the eigenvalues generated from the PAF differed significantly from chance. To do this, we ran 1000 randomized permutations, generated from the raw dataset, and compared the raw mean eigenvalues from the PCA with the 95th percentile permuted eigenvalues, following O'Connor [47]. Raw mean eigenvalues greater than the 95th percentile permuted eigenvalues were considered significant (*p* ≤ 0.05). Overall measures of sampling adequacy were assessed using Kaiser–Meyer–Olkin (KMO) tests and considered satisfactory if greater than 0.5. Sampling adequacy for each task was also assessed using the anti-image correlation matrix and considered satisfactory if KMO was greater than 0.5. Bartlett's test for sphericity was used to determine whether correlations between variables included in the inter-correlation matrix were acceptable (*p* < 0.05). Determinant scores were used to assess multi-collinearity and were considered adequate if greater than 0.00001. To determine the model fit for each combination of tasks included in PAF, observed correlation coefficients were compared to reproduce correlation coefficients generated from the factor model. Models with less than 50% of bivariate residuals greater than 0.05 were considered adequate [48]. Multivariate ANOVA were used to assess whether task performance measures were influenced by sex and body condition (mass/tarsus^3^).

## 3. Results

### 3.1. Does task performance improve across sessions?

With the exception of the Robo-Worm and Detour Reach tasks, which were administered in one trial, subjects' performance improved significantly between the first and final sessions for all tasks (table 2; repeated measures ANOVA): *F*_1, 30_ = 268.73, *p* < 0.001.

**Table 2.** Mean improvement in performance (±s.e.m.): number of correct choices across first and final sessions for each test. Positional Memory performance shows a reduction in errors before locating food reward; Paper Puncture performance shows an increase in technical level achieved; Robo-Worm shows latency to acquire a moving mealworm (sec); Detour Reach shows the number of pecks to the apparatus before acquiring the reward.

tasks	first session	final session	paired *t*-test
Paper Puncture	1.48 ± 0.18	3.48 ± 0.26	*t* = −9.89, d.f. = 30, *p* < 0.001
Robo-Worm	4.20 ± 1.28	n.a.	n.a.
Positional Learning	4.81 ± 0.47	8.13 ± 0.28	*t* = −9.46, d.f. = 30, *p* < 0.001
Positional Memory	4.42 ± 0.33	3.13 ± 0.36	*t* = 3.42, d.f. = 30, *p* = 0.002
Colour Learning 1	4.65 ± 0.29	7.87 ± 0.25	*t* = −8.65, d.f. = 30, *p* < 0.001
Colour Learning 2	5.23 ± 0.25	7.61 ± 0.24	*t* = −7.70, d.f. = 30, *p* < 0.001
Colour Reversal 1	2.23 ± 0.28	5.10 ± 0.33	*t* = −10.82, d.f. = 30, *p* < 0.001
Colour Reversal 2	2.97 ± 0.27	6.10 ± 0.27	*t* = −9.12, d.f. = 30, *p* < 0.001
Detour Reach	16.26 ± 3.82	n.a.	n.a.

### 3.2. Is task performance consistent across tasks?

There was little overall consistency in individual performance across tasks. Four of 36 bivariate relationships showed significant correlations above 0.3 (table 3). Although non-significant, six additional bivariate relations may be included if the coefficients were rounded up to one decimal place, giving a total of 10 of 36 bivariate relationships showing reasonable correlations of at least 0.3. Three significant bivariate relationships correlated positively, while Paper Puncture and Detour Reach performances were inversely related. Interestingly, performance on both motor-related tasks (Paper Puncture and Robo-Worm) were significantly and positively correlated with the first reversal learning task.

**Table 3.** Correlation matrix and *p*-values (one-tailed; in parentheses) of individual performance on each task. Significant values (*p* ≤ 0.05) are presented in italics; non-significant values in roman. Task abbreviations are presented in table 1. Individual task KMO scores, generated from anti-image correlations are presented in square brackets on task diagonals (adequate sample size if greater than 0.5).

tasks	Paper Puncture	Robo-Worm	Positional Learning	Positional Memory	Colour Learning 1	Colour Learning 2	Colour Reversal 1	Colour Reversal 2	Detour Reach
Paper Puncture	[0.67]	0.19 (0.16)	−0.14 (0.23)	0.14 (0.23)	0.20 (0.14)	−0.04 (0.42)	*0.37* (*0.02)*	−0.03 (0.43)	−*0.42* (*0.01*)
Robo-Worm	—	[0.46]	0.01 (0.47)	0.13 (0.24)	0.07 (0.36)	−0.04 (0.42)	*0.41* (*0.01)*	0.21 (0.13)	−0.23 (0.10)
Positional Learning	—	—	[0.56]	−0.26 (0.08)	−0.28 (0.06)	*0.32 (0.04)*	0.01 (0.48)	−0.08 (0.34)	0.11 (0.28)
Positional Memory	—	—	—	[0.62]	0.07 (0.35)	−0.10 (0.31)	0.01 (0.47)	−0.08 (0.34)	−0.20 (0.14)
Colour Learning 1	—	—	—	—	[0.56]	−0.07 (0.36)	0.27 (0.07)	0.27 (0.07)	−0.15 (0.21)
Colour Learning 2	—	—	—	—	—	[0.38]	0.21 (0.13)	0.04 (0.42)	−0.09 (0.31)
Colour Reversal 1	—	—	—	—	—	—	[0.60]	0.13 (0.24)	−0.26 (0.08)
Colour Reversal 2	—	—	—	—	—	—	—	[0.38]	0.28 (0.06)
Detour Reach	—	—	—	—	—	—	—	—	[0.54]

### 3.3. Performance on the full cognitive test battery

When all tasks were included in the correlation matrix they did not appear to suffer from multi-collinearity: determinant = 0.241 (greater than 0.00001 if adequate). However, the overall sample size was low: KMO = 0.53 (greater than 0.5 if adequate). Individual task KMO scores, generated from anti-image correlations, revealed that the sample size for three of the nine tasks was inadequate (presented in square brackets on task diagonals in table 3; should be greater than 0.5). Correlations between variables included in the inter-correlation matrix also failed to reach significance Bartlett's test of sphericity: *p* = 0.41. Twenty-five (69%) of the reproduced correlation coefficients were greater than 0.05 when all tasks were included in the model. Consequently, the coefficients derived from the factor model show a poor fit to the observed correlations.

A PAF including all nine tasks revealed a single factor with an eigenvalue of 1.6, which explained 24.25% of the variance across tasks. However, subsequent parallel analyses, following [47], revealed that this eigenvalue was less than the 95th percentile permuted eigenvalues of 1.76. Hence the explanatory power of the factors was no greater than chance.

### 3.4. Performance on different combinations of six tasks within the full test battery

To determine whether significant factors could be generated by chance from different combinations of tasks within the test battery, we conducted PAFs for all possible combinations that included six of the nine different tasks. We chose to investigate the relationships between six tasks as this provided the largest possible sample size while retaining a reasonably broad selection of different cognitive abilities. Fourteen of these 84 combinations (17%) revealed a significant first factor that was robust to parallel analysis, explaining between 28.63 and 35.54% of individual variation in task performance (table 4). Of these 14 tasks, only one combination (task combination 3) met the assumptions for factorial analysis of sampling adequacy (KMO), covariance (Bartlett's) and no multi-collinearity (determinant), as denoted by an asterisk in table 4. However, if sampling adequacy was considered for each task individually (KMO produced from anti-image correlations of task diagonals; table 5), then four task combinations (task combinations 1, 2, 5 and 9) comprised all tasks with an adequate sample size. More than 50% of the reproduced correlation coefficients were greater than 0.05 for all task combinations. Consequently, the coefficients derived from the factor model show a poor fit to the observed correlations (table 4).

**Table 4.** First factor variance of performance (%) and assumptions for all task combinations in which six of the nine tasks showed significant eigenvalues that were robust to parallel analysis. Requirements of sampling adequacy for each assumption are presented in parentheses. Tasks with asterisk (*) meet all assumptions, excluding reproduced correlation coefficients, in which all tasks failed to meet.

task combination	KMO (greater than 0.5)	Bartlett's (*p* < 0.05)	determinant (less than 0.00001)	reproduced correlations greater than 0.05 (less than 50%)	variance %	eigenvalue	permuted eigenvalue (95%)
1	0.66	0.19	0.49	11 (73%)	35.54	1.47	1.38
2	0.64	0.15	0.47	8 (53%)	35.03	1.46	1.36
3*	0.57	0.04	0.39	10 (66%)	34.57	1.49	1.36
4	0.57	0.14	0.47	10 (66%)	34.55	1.45	1.32
5	0.64	0.20	0.49	9 (60%)	33.90	1.45	1.39
6	0.60	0.10	0.44	10 (66%)	33.38	1.46	1.37
7	0.56	0.12	0.45	14 (93%)	32.80	1.42	1.33
8	0.58	0.12	0.45	12 (80%)	32.68	1.44	1.37
9	0.57	0.27	0.52	10 (66%)	31.83	1.33	1.24
10	0.55	0.34	0.54	8 (53%)	31.44	1.33	1.31
11	0.51	0.28	0.52	10 (66%)	31.23	1.32	1.25
12	0.55	0.20	0.49	11 (73%)	30.28	1.24	1.23
13	0.52	0.51	0.59	10 (66%)	28.87	1.28	1.28
14	0.51	0.35	0.54	8 (53%)	28.63	1.27	1.26

**Table 5.** Measures of sampling adequacy (KMO) for each individual task (produced from anti-image correlations). Tasks with asterisk (*) meet all assumptions of being greater than 0.5.

task combination	Paper Puncture	Robo-Worm	Positional Learning	Positional Memory	Colour Learning 1	Colour Learning 2	Colour Reversal 1	Colour Reversal 2	Detour Reach
1*	0.68	0.62	—	0.60	0.69	—	0.63	—	0.70
2*	0.67	0.64	0.51	—	0.61	—	0.62	—	0.70
3	0.69	0.55	—	—	0.53	—	0.68	0.40	0.54
4	0.62	0.56	—	—	0.61	0.27	0.57	—	0.65
5*	0.65	0.63	0.57	0.59	—	—	0.62	—	0.70
6	0.65	0.59	—	0.64	—	—	0.65	0.44	0.59
7	0.60	0.56	0.50	—	—	0.40	0.58	—	0.63
8	0.65	0.60	0.42	—	—	—	0.65	0.40	0.56
9*	0.62	0.58	0.51	—	0.56	—	0.58	0.53	—
10	0.63	0.54	—	0.40	0.54	—	0.58	0.48	—
11	0.60	0.52	—	—	0.52	0.27	0.53	0.50	—
12	0.66	0.53	0.55	—	0.60	0.45	0.52	—	—
13	0.55	0.55	—	0.50	—	0.36	0.52	0.55	—
14	0.54	0.55	0.48	—	—	0.42	0.52	0.51	—

Salient unrotated factor loadings (greater than 0.4; see [46]) were revealed in three of the nine tasks (Paper Puncture, Robo-Worm, the first Colour Reversal and Detour Reach) for the majority of task combinations (table 6). Salient factor loadings were positive for all tasks but negative for the Detour Reach task. As such, individuals that were fast to learn the motor tasks and rapidly responded to reversed contingencies of a previously learned colour association also took longer to acquire a food item on the Detour Reach task. One other task, Colour Learning 1, also showed generally high factor loadings. The Paper Puncture task, like the Robo-Worm task, was designed to assess motor-related performance and factor loadings for both tasks were positively loaded. Similarly, the Detour Reach task, like the Colour Reversal task, was designed to assess inhibitory control, yet factor loadings for both tasks were inversely loaded. Factor loadings on all other tasks were negligible, suggesting that they did not contribute to subjects' overall performance.

**Table 6.** Matrix of unrotated first factor loadings for all combinations of tasks with significant eigenvalues (*p* ≤ 0.05). Factor loadings in italics (greater than 0.4) are considered salient [46].

task combination	Paper Puncture	Robo-Worm	Positional Learning	Positional Memory	Colour Learning 1	Colour Learning 2	Colour Reversal 1	Colour Reversal 2	Detour Reach
1	*0*.*62*	*0*.*45*	—	0.21	0.32	—	*0*.*62*	—	*−0.56*
2	*0*.*62*	*0*.*42*	−0.12	—	0.35	—	*0*.*63*	—	*−0.54*
3	*0*.*58*	*0*.*46*	—	—	0.34	—	*0*.*69*	0.09	*−0.49*
4	*0*.*59*	*0*.*44*	—	—	0.31	0.10	*0*.*69*	—	*−0.53*
5	*0*.*63*	*0*.*45*	—0.16	0.24	—	—	*0*.*56*	—	*−0.59*
6	*0*.*62*	*0*.*46*	—	0.21	—	—	*0*.*58*	−0.02	*−0.58*
7	*0*.*60*	*0*.*46*	−0.01	—	—	0.09	*0*.*65*	—	*−0.55*
8	*0*.*62*	*0*.*46*	0.11	—	—	—	*0*.*62*	0.01	*−0.55*
9	*0*.*45*	*0*.*46*	−0.16	—	*0.41*	—	*0*.*73*	0.27	—
10	*0*.*44*	*0*.*49*	—	0.11	0.36	—	*0*.*77*	0.24	—
11	*0*.*41*	*0*.*45*	—	—	0.34	0.11	*0*.*87*	0.24	—
12	*0*.*45*	*0*.*42*	−0.01	—	0.32	0.08	*0*.*88*	—	**—**
13	0.39	*0*.*48*	—	0.08	—	0.13	*0*.*89*	0.17	—
14	0.37	*0*.*45*	0.01	—	—	0.14	*0*.*94*	0.17	—

### 3.5. Is task performance related to sex or body condition?

Two individuals died before we could determine their sex and hence were excluded from the following analyses (females *N* = 11; males *N* = 18). We found no effect of sex (*F*_9, 9_ = 0.39, *p* = 0.91) or body condition (*F*_63, 105_ = 0.97, *p* = 0.54) on subjects' performance across tasks. No interactions between sex and body condition were observed (*F*_18, 20_ = 0.75, *p* = 0.73).

## 4. Discussion

Individuals were rarely consistent in their performance across multiple tasks, both within similar and across different cognitive domains. Yet, when different subsets of six of the nine tasks were included in factor analyses, a single factor, robust to parallel analysis, could be extracted in 14 of the 84 possible task combinations. Performances on the two motor tasks and the first Colour Reversal showed strong positive loadings on the majority of 14 combinations, while the Detour Reach task showed strong negative loadings. These findings suggest that birds with good motor skills rapidly solved a Reversal Learning task, but showed poor Detour Reach performance. Nonetheless, we found no evidence that a robust single factor comprised a broad array of cognitive domains with strong positive loadings. We therefore found no support for either domain-general or domain-specific cognitive abilities in pheasants. However, we did find bivariate relationships between performances in apparently unrelated pairs of cognitive domains, suggesting some linkage between disparate cognitive task performances. While this is a reasonably large study among non-human animals, our findings may however be constrained by the small (and partial) sample size, and hence should be interpreted with caution. Importantly, we demonstrate how intra-individual variation in performance across tasks can influence single factor explanations of cognitive processes. Our findings therefore highlight the importance of conducting multiple tests within specific domains to ensure that individual performance reflects putative cognitive abilities.

Tasks with factor loadings greater than 0.4 have been considered salient [46], with higher loadings reflecting greater task complexity [9]. Like Shaw *et al.* [22], we found strong positive component loadings on a Colour Reversal task. However, in contrast to Shaw *et al*. [22], Detour Reach performance had negative loadings (table 6). Individuals that show greater capacities for reversal learning have been considered more cognitively flexible than individuals that performed poorly at these tasks [37,38]. However, our findings suggest that individuals that performed well on a reversal learning task made more redundant errors when accessing a worm behind a clear Perspex barrier. Our findings are therefore conflicting as both Detour Reach and reversal learning tasks have been considered to involve similar processes that reflect capacities for inhibitory control [35,49].

Performance on the two motor-related tasks had strong positive loadings, while the two positional tasks showed weak loadings. While salient negative component loadings on motor-related problems have been found in a barrier removal task presented to male satin bowerbirds [50], motor-related task performance has also been considered a poor measure of cognitive ability; perhaps due to individual differences in prior motor-related experiences [22]. Yet, unlike previous studies that test wild birds on motor-related tasks [21,22,31,34], our subjects were raised under standardized conditions and hence experienced similar motor-related interactions during their development. Hence, individual differences in motor performance in our study may more accurately reflect cognitive performance on a novel task, rather than being confounded by prior experience. The positive loadings of motor-related performance and the negative loadings on the Detour Reach task in the current study may reveal a possible trade-off in foraging strategies. For example, some individuals may rapidly act to extract or capture food but show greater perseveration of unrewarded actions, whereas other individuals that are less successful at capturing or extracting food items may show better inhibitory control and hence wait until their chances of success are more favourable. Capacities for cognitive and motor function may therefore be mediated by competition for limited neural resources, as has been revealed in humans suffering from traumatic brain injury [51].

Factor loadings for the Positional Learning, Positional Memory and Colour Learning tasks and the second Colour Reversal task were low, and hence did not notably contribute to an individual's overall performance. Low factor loadings on positional tasks in pheasants contrast with the high loadings observed in North Island robins on similar spatial tasks [22]. The different contributions of factor loadings for positional tasks on general cognitive performance between these species may be due to different cognitive specializations. For example, caching species, such as North Island robins, rely on remembering multiple spatial locations to facilitate the retrieval of previously stored food items [52]. Non-caching birds, such as pheasants, may not be challenged with such tasks. As such, neuroanatomical adaptations associated with memory likely differ between caching and non-caching birds [53].

Subtle variations in the types of tasks included in a given test battery resulted in marked differences in whether interpretations of a single factor could be used to infer capacities for domain-general cognition. Accordingly, parallel analysis generated a significant single factor in 14 of 84 combinations (17%) that included six of the nine different tasks. When a robust single factor was extracted, it typically explained 28–35% of variation of overall performance on the test battery. These findings are similar to values obtained in studies of humans [8], non-human primates [13], mice [9] and other avian species [21,22,31] which concluded that a general cognitive ability ‘*g*’ underlies performance on cognitive test batteries [5]. Yet, bivariate relationships between tasks were rarely significant in the current study. Consequently, intra-individual variation in task performance leads to only a subset of tasks contributing salient factor loadings. Only one of 84 task combinations (1%) met the general assumptions required for factor analysis. All task combinations showed a poor model fit, suggesting that the observed correlation coefficients from the raw data differed from those generated by the factor analysis. While our sample size is reasonably large compared with many other similar studies in birds [21,22,31,32,54], it remains small for factor analysis. Hence findings based on analyses of 31 subjects on nine tasks, providing a ratio of under four, are likely to be constrained by low power. A more representative sample of 45–90 subjects, yielding a ratio of 5 : 1–10 : 1 would be more appropriate for such analyses. However, analyses of performances on the six tasks provide a ratio greater than five, which remains acceptable. Consequently, we found no evidence to suggest that the performances of pheasant chicks on our cognitive test battery were governed by a general capacity comprised of several disparate cognitive processes. Instead, our findings reveal marked inconsistencies in performance, not only across different tasks representing different domains, but also within similar tasks.

Our test battery comprises a variety of novel variants of domain-specific tasks that we considered related to one another (e.g. two colour learning tasks, two reversal learning tasks and two motor tasks), as well as distinct tasks that were considered to assess performance across different, unrelated, cognitive domains (e.g. Positional Memory and motor performance). Previous studies have used similar approaches, revealing that human children differ from non-human great apes on social but not physical cognition tasks [55]. By contrast, our findings revealed little consistency in task performance, not only within but also across different cognitive domains. Inconsistent performances were exemplified among novel variants of the colour learning and reversal learning tasks, which differed only in cue colour. These findings highlight that individual performance on one task may not always relate to performance on similar tasks, even when using subtle variations of colour cues, or across similar learning paradigms involving either colour learning or reversal discriminations. However, weak bivariate correlations are frequently reported in similar studies that compare subjects' performance across different tasks; for example, only 4 of 15 [22], 8 of 15 [21] and 2 of 15 [31] tasks showed bivariate relationships above 0.3 (but see [32]). In contrast to humans and non-human apes, which demonstrate performances that cluster across multiple exemplars of similar tasks within specific cognitive domains [26,55], non-human animals that are tested in the wild are often presented with single-task exemplars that are considered to reflect their capacities within that particular domain [21,22,31]. Our findings therefore highlight the importance of conducting multiple tests to ensure intra-individual consistency in performance both within and across cognitive domains.

It remains possible that our test battery failed to capture general learning performance in pheasants, due to our testing procedures, or test battery design. Only 31 of 200 birds completed all 290 trials on the nine different tasks. While all birds had equal opportunity, free from competition, to enter the testing chamber and engage with each task, such high attrition suggests that birds that participated may differ from those that failed to participate. Consequently, our study may have comprised a biased subsample of the population. Indeed, we have found in previous studies on pheasants that a number of non-cognitive, motivational, traits can influence participation on cognitive tasks [40]. Hence, more exploratory pheasants, or those that were particularly food motivated, may result in a self-selecting sample, by voluntarily participating in the tasks. These individuals may then represent either end of the extremes of the distribution of general cognitive ability. At the one end, individuals that are more exploratory may show greater cognitive ability. Exploratory behaviours in mice, for example, covary with general learning ability [19]. Object exploration has also been considered an important trait associated with cognitive flexibility in birds, such as the kea [56]. At the other end of the spectrum, individuals that are highly food motivated may show poor cognitive performance, as has been demonstrated in other species of birds, such as the North Island robin [57]. As such, pheasants that are more exploratory and food motivated may be more likely to participate in appetitive food-related tasks. Yet, exploratory and highly food motivated individuals may inadvertently represent subjects from both tails of a cognitive distribution, hence ensuring that no general factor could be detected. However, we consider this explanation unlikely as we have found in a previous study that pheasants which rapidly acquired a freely available mealworm (Baseline Worm), were more likely to participate in a cognitive test [40]. Moreover, while we might expect differences in growth rates in response to an individual's sex or body condition to influence their food motivation, we found no effect of sex or body condition on task performance in the current study. The alternative to our use of voluntary participation with its attendant attrition of subjects is to obtain performance measures from subjects' forced participation on non-appetitive tasks. However, while such tasks may illuminate whether performance is related to food motivation, such procedures may also confound results, as stress (e.g. induced by forcing participation) can have detrimental influences on cognitive performance [58]. Forced participation may also be difficult to achieve in studies conducted in the wild, making comparisons between forced and appetitive procedures difficult to interpret.

Our test battery failed to capture performances that had strong positive loadings on multiple tasks across multiple domains. This finding may suggest that pheasants lack capacities for general intelligence. Yet, there also remains the possibility that the construction of our test battery included tasks that shared no general influence. As such, we cannot conclude that pheasants lack capacities for domain-general intelligence, but rather capacities for such processes may not be revealed in every cognitive test. The tests included in our battery may have also lacked ecological validity, been too difficult, or the cognitive underpinnings unclear. However, we consider this unlikely as improvements in performances on all tasks with multiple trials suggest that learning is invoked (at the population level). Finally, it remains possible that our findings reflect a relatively high contribution of non-cognitive influences (or simply noise) that we did not or cannot identify. As such, we present a preliminary investigation into the performances of pheasants on a cognitive test battery comprised of tasks that are frequently used to assess cognitive abilities of non-human animals.

We demonstrate that subtle variations in the types of tasks included in a given test battery can influence interpretations of whether a single factor reflects capacities for domain-general cognition. Consequently, acceptance of a domain-general intelligence, especially in animal studies, should be tempered by the knowledge that the chance of extracting a single component that we may conceive of as ‘*g*’, even if robust to parallel analysis, may be highly susceptible to the exact composition of the test battery used. Even when a robust single factor summarized subjects' performance on test batteries comprised of different subsets of tasks, we found no evidence that these represented a general cognitive ability. Accordingly, few of our tasks that represented different cognitive domains showed salient loadings on a single factor. Consequently, intra-individual variation in performance on tasks administered in our study highlights the importance of using multiple tests to ensure robust measures of cognitive abilities within particular domains are obtained.

## Supplementary Material

Dataset

## Data accessibility

All data used in this study can be accessed in the electronic supplementary materials.
